# COVID-19: efficacy of prehospital pulse oximetry for early detection of silent hypoxemia

**DOI:** 10.1186/s13054-020-03185-x

**Published:** 2020-08-13

**Authors:** Valentina Quaresima, Marco Ferrari

**Affiliations:** Department of Life, Health and Environmental Sciences, University of L’Aquila, Via Vetoio, 67100 L’Aquila, Italy

We read with interest the research letter published in *Critical Care* by Jouffroy et al. who suggest the utility of prehospital pulse oximetry as a red flag for early detection of silent hypoxemia in COVID-19 patients [1]. Fingertip pulse oximeters are one of the most widely used medical standard monitoring tools to assess oxygenation and respiratory function in patients. In fact, a pulse oximeter noninvasively measures arterial blood oxygen saturation (SpO_2_, %), and it represents an accessible tool that can be easily used by patients, physicians, and prehospital healthcare providers. However, it is important to ensure that the used pulse oximeter has a high accuracy, especially when SpO_2_ is lower than 90%. The accuracy criteria for the pulse oximeter equipment are provided in the International Standard ISO 80601-2-61 [2], and the U.S. Food and Drug Administration (FDA) only permits pulse oximeters that meet these criteria [3]. According to the ISO 80601-2-61, the SpO_2_ accuracy of a pulse oximeter should be assessed through a controlled desaturation study by comparing the SpO_2_ measurements with the gold standard measurements of blood arterial oxygen saturation (SaO_2_) obtained by a CO-oximeter. The prices for home pulse oximeters range from $20 to $80, and not all pulse oximeters are built with the same performances and the required FDA’s accuracy. Few of them have the FDA’s approval.

A recent study on the accuracy of six low-cost pulse oximeters, not cleared by the FDA, has demonstrated highly inaccurate readings, even if some of them surprisingly performed likewise the expensive clinical pulse oximeters during voluntary hypoxia [4].

Some COVID-19 patients suddenly develop the condition called “silent hypoxia,” during which they still look and feel comfortable, but their SpO_2_ is perilously low. This happens to patients either in the hospital or at home. Low SpO_2_ may indicate severe COVID-19-related pneumonia, requiring a ventilator. A useful practical guidance for the correct use of pulse oximetry for monitoring patients with COVID-19 at home have been very recently published [5]. SpO_2_ self-monitoring by patients with non-severe COVID-19, discharged from the emergency department or an outpatient testing center, is an essential way to identify patients needing to return to the hospital for a further evaluation.

However, considering the largely unregulated low-cost pulse oximetry market, physicians should pay attention to incorporating SpO_2_ data, obtained from pulse oximeters not cleared [3] by the FDA, the European Medicine Agency or other regulatory agencies, for making medical decisions.

## Authors’ response

### Reply to Quaresima et al.: “COVID-19: efficacy of prehospital pulse oximetry for early detection of silent hypoxemia”

Romain Jouffroy, Daniel Jost, Bertrand Prunet

Dear Editor,

We thank Quaresima et al. [6] for their interest and relevant comments about the early detection of silent hypoxemia in COVID-19 patients based on prehospital pulse oximetry [1]. We agree that fingertip pulse oximeters may only provide approximate measurements of arterial blood oxygen saturation and that many intrinsic and extrinsic factors influence pulse oximetry values [6, 7] beyond the preanalytical conditions required for certification [8]. One particularity of COVID-19-related acute respiratory failure was that, despite low blood oxygen concentration, the respiratory rate frequently remained close to normal, unlike in other acute respiratory failure etiologies [9]. Our observational study was not designed to assess pulse oximetry for medical decision making but was intended to detect “silent hypoxemia” in suspected COVID-19 patients. Furthermore, the Paris Fire Brigade basic life support teams used a device complying with the European Union’s Medical Device Regulation of 2017. Quaresima et al. remind us that rescuers must continuously be aware of the technical limitations of the equipment they use in their daily practice.

## Data Availability

Not applicable.

## References

[1] Jouffroy R, Jost D, Prunet B (2020). Prehospital pulse oximetry: a red flag for early detection of silent hypoxemia in COVID-19 patients. Crit Care.

[2] International Standard (2017). ISO 80601-2-61:2017. Medical electrical equipment — Part 2–61: Particular requirements for basic safety and essential performance of pulse oximeter equipment.

[3] Pulse Oximeters. U.S. Department of Health and Human Services, Food and Drug Administration. Premarket Notification Submissions [510(k)s]: Guidance for Industry and Food and Drug Administration Staff. Published Mar 4, 2013.

[4] Lipnick MS, Feiner JR, Au P, Bernstein M, Bickler PE (2016). The accuracy of 6 inexpensive pulse oximeters not cleared by the Food and Drug Administration: the possible global public health implications. Anesth Analg.

[5] Luks AM, Swenson ER. Pulse oximetry for monitoring patients with COVID-19 at home: potential pitfalls and practical guidance. Ann Am Thorac Soc. 2020. 10.1513/AnnalsATS.202005-418FR. 10.1513/AnnalsATS.202005-418FR. Published online ahead 1 of print, 2020 Jun 10. 10.1513/AnnalsATS.202005-418FR PMC7462317 32521167

[6] Quaresima V, Ferrari M. COVID-19: efficacy of prehospital pulse oximetry for early detection of silent hypoxemia. Crit Care. 2020;XXXX. 10.1186/s13054-020-03185-x PMC7424128 32791979

[7] Rich K (2001). Transcutaneous oxygen measurements: implications for nursing. J Vasc Nurs.

[8] Dagher G, Becker KF, Bonin S (2019). Preanalytical processes in medical diagnostics: new regulatory requirements and standards. New Biotechnol.

[9] Braman SS (1995). The regulation of normal lung function. Allergy Asthma Proc.

